# The social formation of fitness: lifetime consequences of prenatal nutrition and postnatal care in a wild mammal population

**DOI:** 10.1098/rstb.2022.0309

**Published:** 2023-08-14

**Authors:** E. I. K. Vitikainen, M. Meniri, H. H. Marshall, F. J. Thompson, R. Businge, F. Mwanguhya, S. Kyabulima, K. Mwesige, S. Ahabonya, J. L. Sanderson, G. Kalema-Zikusoka, J. I. Hoffman, D. Wells, G. Lewis, S. L. Walker, H. J. Nichols, J. D. Blount, M. A. Cant

**Affiliations:** ^1^ Centre for Ecology and Conservation, University of Exeter, Penryn, Cornwall, TR10 9FE, UK; ^2^ Organismal and Evolutionary Biology, University of Helsinki, Helsinki, PO Box 65, 00014 Finland; ^3^ Centre for Research in Ecology, Evolution and Behaviour, University of Roehampton, Roehampton Lane, London SW15 5PJ, UK; ^4^ Banded Mongoose Research Project, Queen Elizabeth National Park, PO Box 66 Lake Katwe, Kasese District, Uganda; ^5^ Conservation Through Public Health, PO Box 75298, Uringi Crescent Rd, Entebbe, Uganda; ^6^ Department of Behavioural Ecology, University of Bielefeld, Bielefeld, Konsequenz 45, 33619, Germany; ^7^ Department of Biosciences, Wallace Building, Swansea University, Singleton Park, Swansea SA2 8PP, UK; ^8^ Chester Zoo Endocrine Laboratory, Endocrinology, Science Centre, Caughall Road, Upton-by-Chester, Chester, CH2 1LH, UK; ^9^ German Primate Center, University of Goettingen, Kellnerweg 4, 37077 Göttingen, Germany

**Keywords:** early life effects, social evolution, evolution of parental care, cooperative breeding, fetal programming

## Abstract

Research in medicine and evolutionary biology suggests that the sequencing of parental investment has a crucial impact on offspring life history and health. Here, we take advantage of the synchronous birth system of wild banded mongooses to test experimentally the lifetime consequences to offspring of receiving extra investment prenatally versus postnatally. We provided extra food to half of the breeding females in each group during pregnancy, leaving the other half as matched controls. This manipulation resulted in two categories of experimental offspring in synchronously born litters: (i) ‘prenatal boost’ offspring whose mothers had been fed during pregnancy, and (ii) ‘postnatal boost’ offspring whose mothers were not fed during pregnancy but who received extra alloparental care in the postnatal period. Prenatal boost offspring lived substantially longer as adults, but postnatal boost offspring had higher lifetime reproductive success (LRS) and higher glucocorticoid levels across the lifespan. Both types of experimental offspring had higher LRS than offspring from unmanipulated litters. We found no difference between the two experimental categories of offspring in adult weight, age at first reproduction, oxidative stress or telomere lengths. These findings are rare experimental evidence that prenatal and postnatal investments have distinct effects in moulding individual life history and fitness in wild mammals.

This article is part of the theme issue ‘Evolutionary ecology of inequality’.

## 1. Introduction

Family life has evolved in a very wide range of taxa because of the advantages that parental and alloparental (i.e. helping) investment can provide for developing offspring [1,2]. Offspring that receive more resources or care during development often survive better [3–5], attain a larger adult size [5–7], breed earlier [8] and/or enjoy enhanced reproductive success as adults [5,7,9–11]. Classic life-history theory predicts that parents and helpers who experience lower opportunity costs of caring, or those whose help results in a greater boost to offspring fitness, should allocate more investment per offspring or brood over their lifetime [12–15]. These and other predictions concerning optimal allocation of parental investment have received widespread empirical support (reviewed in [1,16]).

Much less attention has focused on a second crucial aspect of a parent or helper's investment decision: the scheduling of investment over the course of development. Most existing models of parental investment consider optimal allocation to a single stage of development or average allocation per offspring (reviewed in [15,17]). In humans and many other animals, however, parents provide resources over an extended period which includes distinct phases of gestation, postnatal nursing and juvenile provisioning. Resources allocated to offspring at one stage of development may affect the capacity of offspring to respond to investment at later stages, so that investments at each stage may combine or interact in non-additive ways [18,19]. For example, resources allocated early in life may be lost entirely unless they are followed up with investment at later stage: this is the case for example, where there are minimum thresholds of investment required to survive at one or more developmental stages [13,14,18,20]. The optimal schedule of investment for parents and helpers should thus depend on how investment at each stage of development affects the fitness benefits to offspring of investment at later stages and subsequently across the adult lifespan.

The optimal scheduling of developmental investment for humans is the focus of economic studies of ‘skills formation’ [18,19,21] and ‘health capital’ [22–25]. These studies address the question of how investment during childhood influences key attributes of adults such as their lifetime earnings and health outcomes, and what stages of development should be targeted for positive intervention to have the maximum health or economic benefits to adults [26]. Theoretical models of skill formation break the developmental period up into multiple stages and specify the nature of developmental linkage or ‘complementarity’ between stages, that is, how investment at one stage influences the robustness and responsiveness of offspring at the next developmental stage [19]. A general finding of these studies, both theoretical and empirical, is that targeting investment at earlier stages of development is a more cost effective way to maximize adult skills or health than attempting to remediate later on [18,21,26].

It is unclear whether this principle that ‘earlier investment is more efficient’ extends to the prenatal period. In human health studies, optimal development is proposed to require a match between the prenatal and postnatal nutritional environments (the ‘developmental mismatch’ hypothesis [27–29]). Hence, large offspring born into a resource-scarce environment and small offspring born into a resource-rich environment are both predicted to develop sub-optimally in terms of maximizing fitness [27,30]. Data in humans suggest that both maternal under-nutrition and over-nutrition during pregnancy are associated with later poor health outcomes for offspring, providing some support for this prediction [31,32]. In addition, experiments on animal models show causally that mismatch between the quality and stressfulness of prenatal and postnatal environments leads to negative fitness outcomes [33,34]. However, several studies of wild vertebrate populations have failed to find support for the developmental mismatch hypothesis (e.g. [35–38]). To our knowledge, there are no experimental data from wild mammals to understand the relative importance of prenatal versus postnatal investment, and complementarity between them, in shaping growth, physiology, life history and fitness. Such information is important to understand whether mammalian development is shaped by natural selection to respond optimally to schedules of investment set by parents or, in cooperative animal societies, parents and alloparents of varying degrees of kinship.

Here, we report a long running field experiment in a wild cooperative mammal, the banded mongoose (*Mungos mungo*), which we use to test the lifetime physiological and life-history consequences of varying investment received by offspring in prenatal versus postnatal periods of development. In this species, multiple adult females in each group (median = 5) synchronize birth to the same morning, producing a joint litter of 5–25 offspring (median 12) that are guarded and suckled underground or at the entrance to the den for the first month of life, apparently without any discrimination according to maternity [39,40]. When they are one month old, offspring emerge from the den and start to accompany the group on foraging trips, during which they are escorted and provisioned on a one-to-one basis by particular male or female adults called escorts [41–43]. Most pups have a single escort until they reach nutritional independence at 10 weeks old, but some pups spend a small percentage of their time with additional escorts [42]. Escorts provide insect prey and small vertebrates to the pup in their care and guard and defend it from predators. Escort-pup pairs are no more closely related to each other than expected by chance [44]. Pups who spend more time in close association with their escort during development receive more food, grow faster and attain larger body size at adulthood compared to pups that do not have a close escorting relationship [5].

In a previous paper, we described the first results from our experiment to provision half of the pregnant females in each group with high-quality resources (cooked egg) throughout the latter half of gestation, leaving the other half of pregnant females as matched controls [45]. We found that mothers who received extra food in pregnancy gave birth to offspring which were heavier than the offspring of non-fed mothers, as we expected. However, subsequently, these fed mothers targeted extra care and investment at the (smaller) offspring of unfed mothers in the escorting period, whereas their own offspring were escorted by unfed mothers and other group members at ‘typical’ levels. Thus our provisioning experiment, coupled with the targeted helping responses of fed mothers in the postnatal period, resulted in two categories of offspring in experimental litters:

(i) ‘prenatal boost’ (PRE) offspring, whose mothers received extra food during pregnancy, but who received ‘typical’ levels of investment during the escorting period; and
(ii) ‘postnatal boost’ (POST) offspring, whose mothers were not fed in pregnancy, but who received supernormal levels of investment by escorts in the escorting period.

In this paper, we follow the long-term fate of these two categories of offspring and compare them with offspring from unmanipulated breeding attempts. Specifically, we have two main objectives. First, we aim to test the effect of the manipulation on four key life-history metrics: (i) weight at 1 year, (ii) age at first reproduction, (iii) adult survival, and (iv) lifetime reproductive success (LRS). Second, we aim to investigate three proposed physiological mechanisms underlying these key life-history metrics: (i) telomere length, (ii) glucocorticoid levels, and (iii) markers of oxidative stress. There is evidence from a wide range of vertebrates that these physiological mechanisms are implicated in life-history trade-offs (e.g. [46–48]). However, it is usually difficult to establish the causal role of these mechanisms, particularly in wild populations [49]. Our experimental design provides a unique opportunity to compare the physiology of offspring born on exactly the same day in the same group and territory, exposed to the same or similar predation risks and social stressors, yet subject to very different schedules of prenatal and postnatal investment.

## 2. Material and methods

### (a) Study population

Data were collected from a population of wild banded mongooses on the Mweya Peninsula, Queen Elizabeth National Park, Uganda (0°12′ S, 29°54′ E). Detailed life-history data on this population has been collected continuously since 1995 [40,50]. Typically, our study population consists of 10–12 social groups, that are visited every 1–3 days to record group composition, life-history and behavioural data, and for collection of faecal samples and weight data. Most individuals are trained to step onto a portable electronic balance in return for a small milk reward and are weighed weekly in the field before morning foraging. Banded mongooses are cooperative breeders where births are synchronized within the social groups, with on average five females (mean ± s.d. = 5.0 ± 2.6, *n* = 84 litters; [45]) giving birth to a communal litter which is then jointly cared for by the group members [40]. After emergence from the den, most pups form one-to-one relationships with helpers—termed escorts—that feed and protect them [42,43,51]. During the escorting period, groups are visited daily to identify pup-escort pairs and quantify the strength and fidelity of the escorting relationship. We calculated escorting index [42,44] as the proportion of observation sessions in which the pup in question was classified as being escorted, i.e. spent over half of the focal observation session within 0.5 m of an adult. In practice, although pups can in principle associate with many adults, strong relationships that predict pup fitness as adults are a one-to-one bond in which pups rarely receive help from other adults, and adults rarely give help to other pups. Individuals in the population are identified using unique shave markings on their back, and passive integrated transponder tags (TAG-P-122IJ, Wyre Micro Design Ltd., UK) inserted under the skin on the scruff of the neck. To enable tracking the groups, one to two individuals in each group are fitted with radio collars (Sirtrack Ltd, Havelock North, New Zealand) with a 20 cm whip antenna (Biotrack Ltd., Dorset, UK). Individuals within the population are trapped every 3–6 months, using box traps (67 × 23 × 23 cm; Tomahawk Live Trap Co., Tomahawk, WI, USA) and anaesthetized using isoflurane (for details, see [52,53]) for morphometrics and collection of blood samples. Animals are released back to their group within 2 h; no animal has ever died or become sick as a result of trapping and anaesthetization. For further information, see the ethics statement.

### (b) Experimental provisioning of pregnant females

We manipulated prenatal condition in eight groups between August 2013 and April 2017 as described in detail in Marshall *et al.* [45]. Gestation lasts on average 62 days [54] and groups containing pregnant females are visited daily for accurate detection of birth dates from change of body shape of the females. During breeding attempts we provisioned half of all pregnant females in 34 communal breeding attempts involving 101 fed and 97 unfed mothers. Females were fed an average of 50 g cooked egg per day (bird eggs are a natural high value component of the diet). The amount of egg received by fed females each day (0, 50, 100 g) and the time of day she received the egg (morning or afternoon) was randomized to ensure that females could not predict the amount and timing of provisioning, which might have influenced their natural foraging behaviour. To avoid carry-over effects of provisioning, experimental litters were followed by a ‘rest’ litter in which no pregnant females were provisioned. The manipulation resulted in exactly 50 pups from fed females, and 50 pups from unfed females that survived to emerge from the den at the age of one month. These are the pups that are the focus of the current investigation. For more details of the experiment see [45].

In our previous paper [45], we reported the impacts of this experiment on patterns of cooperative care and pup weight up to the age of three months. We briefly summarize the most relevant findings here. Females were captured 3–4 weeks after oestrus and pregnancy confirmed via ultrasound. Provisioning began 2–4 days after pregnancy was confirmed and continued for an average of 24 days (± 9 days, mean ± s.d.). Median number of breeding females was 6 (range 2–10), and median adult group size was 22 (range 6–34). In each experimental litter, approximately half of breeding females (median = 0.5) were assigned to be fed, the other half were left as matched within-group controls. Fed mothers gained significantly more weight over their pregnancy compared to unfed mothers (per cent change in weight fed versus unfed mothers: 24.1 ± 4.5 versus 15.1 ± 3.7) and gave birth to heavier offspring (estimated birth weight of offspring of fed versus unfed mothers: 164.9 ± 3.5 g versus 142 ± 3.2 g). After pups emerged from the den, fed mothers escorted pups at significantly higher levels than unfed mothers (and males) in the same breeding attempt, and more than mothers in unmanipulated breeding attempts. Moreover, they targeted this extra escorting effort at the pups of unfed mothers rather than their own offspring. Mothers that were fed invested twice as much care in offspring from unfed mothers compared to offspring from fed mothers (note that this ‘levelling up’ behaviour is not seen in unmanipulated litters, where there is much less disparity in condition between carers). The offspring escorted by fed mothers were related to them by median relatedness 0.09 (interquartile range (IQR) = −0.008 to 0.25), adding to previous evidence that mothers cannot discriminate their own offspring within synchronously born communal litters [39,45,55]. As a result of extra postnatal investment by fed mothers, the offspring of unfed mothers received more escorting overall, were fed at higher rates and grew faster than the offspring of fed mothers in the same litter. The offspring of fed mothers by contrast received ‘typical’ levels of escorting and grew at the same rate as unmanipulated pups [45].

**Figure 1.**
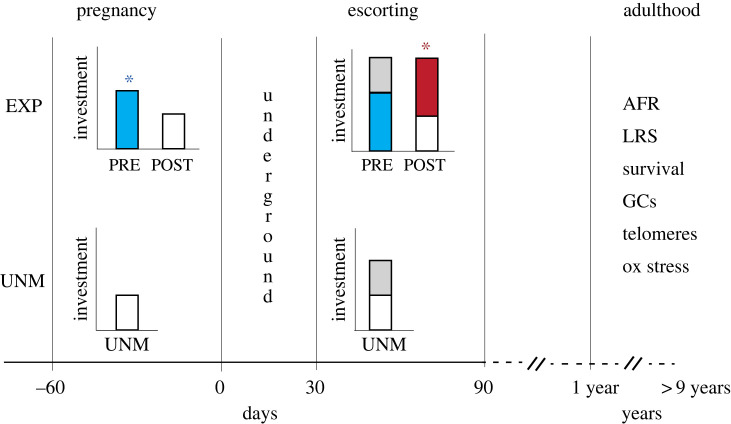
Schematic of developmental stages and the experimental design. The *x*-axis shows the timeline of the key developmental periods; bar graphs represent investment received during pregnancy and escorting. All adult females in each group enter oestrus around the same time and gestate offspring for two months. Offspring are conceived at *approximately* day 60, born synchronously on day 0, remain underground in the den until day 30 and are escorted by helpers until nutritional independence at day 90. Adulthood is reached at 1 year. In experimental (EXP) litters (top row), half the pregnant females in each group were fed high nutrient resources (50 g egg d^−1^) during pregnancy (blue asterisk). As a consequence of provisioning the offspring of fed females (PRE offspring) received more investment *in utero* (blue bar) but went on to receive ‘normal’ levels of escorting in the postnatal period (grey stacked bar). The offspring of unfed females received ‘normal’ levels of prenatal investment (white bar) but went on to receive ‘supernormal’ levels escorting in the postnatal period (red bar and asterisk), which levelled initial inequalities in birth weight and equalized survival to adulthood [45]. The current analysis compared the life-history patterns and physiology of the experimental groups, (PRE versus POST) up to adulthood (1 year) and across the lifespan (max lifespan in this study = 9 years), together with life-history data on offspring from unmanipulated breeding attempts (UNM offspring; white and grey bars). AFR, age at first reproduction; LRS, lifetime reproductive success; GCs, faecal glucocorticoids; ox stress, oxidative stress markers: malondialdehyde, protein carbonase, superoxide dismutase.

Experimental provisioning of pregnant females thus resulted in the two categories of offspring described in the introduction: PRE offspring, who received prenatal investment followed by typical levels of postnatal care or food; and POST offspring, who received typical levels of prenatal investment followed by supernormal levels of postnatal care and food. Finally, for certain analyses we compared life-history patterns observed in experimental litters (PRE and POST) with those in unmanipulated litters measured before the provisioning experiment began. The unmanipulated litter data was composed of 40 litters in 11 groups, measured across an 8 year period from 2009 to 2017. Of the 72 identified mothers that contributed to the dataset, 13 contributed to all three categories of offspring; 25 out of 72 mothers had pups in both unmanipulated and experimental litters; and 16 out of 42 mothers that contributed to experimental litters had pups in both PRE and POST categories. Figure 1 shows a schematic of the experimental design and the resulting categories of offspring.

### (c) Physiological sampling

Pups were trapped within two weeks of emergence from the den (between 30 and 50 days of age) and anaesthetized using isoflurane. They were then weighed, measured and marked using commercially available blonde hair dye (L'Oreal, UK). An approximately 2 mm skin sample was collected from the tail tip for genetic assignment of parentage using a panel of 43 polymorphic microsatellite markers. For further details of the set-up, see [45].

#### (i) Collection of blood samples

Blood (volume 100–500 µl) was collected from the jugular vein using a 25G needle and syringe, and transferred to a 3 ml EDTA BD Vacutainer. The whole blood sample was centrifuged at 2000*g* for 4 min at 4°C (Spectrafuge mini centrifuge, Sigma Aldrich, UK) to extract plasma, which was frozen for analysis of malondialdehyde (MDA) and protein carbonyls (PCs). Samples of red blood cells (RBC) were frozen for later analysis of glutathione (GSH) and superoxide dismutase (SOD) and buffy coat containing white blood cells (WBC) frozen for analysis of telomere length. All samples were snap-frozen in liquid nitrogen within 10 min of collection, transported to the laboratory in Cornwall, UK, in a cryogenic shipper (Taylor-Wharton CX100, Jencons, UK) and stored at −80°C until analysis.

#### (ii) Quantification of oxidative stress markers

Laboratory analyses were performed blindly with respect to sample identity and the experimental design. All steps were conducted on ice to minimize oxidation. All chemicals were high performance liquid chromatography (HPLC) grade, and chemical solutions were prepared using ultra-pure water H_2_O MQ (Milli-Q Synthesis; Millipore, Watford, UK). Only samples collected less than 1 year prior to the first laboratory analyses were selected for quantification and further assays were conducted within 2 years of collection, except for SOD which had to be re-quantified owing to equipment failure (time since collection: MDA: 314 ± 106 days; PC: 362 ± 83 days; SOD: 800 ± 59 days; GSH: 528 ± 39 days). However, time since collection did not significantly influence the level of any of the markers (MDA: *F* = 0.40, *p* = 0.52; PC: *F* = 2.18, *p* = 0.14; SOD: *F* = 1.68, *p* = 0.20; GSH: *F* = 0.08, *p* = 0.77).

#### (iii) Malondialdehyde

Plasma MDA was determined using HPLC with a fluorescence detector (Agilent 1000). We followed the method in Nussey *et al*. [56] with some modifications. Details about the method can be found in [48]. Repeatability (corresponding to intraclass correlation coefficient, *sensu* [57]) computed for 22 duplicate samples was 81.8%.

#### (iv) Protein carbonyls

Plasma PCs were measured using a colorimetric assay (Protein Carbonyl Assay Kit, Cayman Chemical Company), using a molecular devices plate reader (Spectramax M2; Molecular Devices, USA). Owing to limitations in the amount of plasma available as well as the high protein content of samples, 50 µl of plasma was used in the sample and control tubes instead of the 200 µl recommended in the kit instructions. Carbonyl content in samples was expressed in nmol mg^−1^ protein in the controls; repeatability computed for 88 duplicate samples was 87.9% [58].

#### (v) Superoxide dismutase

We assessed SOD activity (U ml^−1^) in RBC samples using the Cayman chemicals SOD assay kit (Cayman Chemical Company, USA). RBC samples were diluted in a 1 : 10 w/v solution using ice cold H_2_O MQ and then centrifuged at 10 000*g* for 15 min at 4°C. The supernatant was collected and further diluted 1 : 100 with sample buffer for quantification. The quantification is based on the detection of superoxide radicals generated by xanthine oxidase and neutralized by SOD, using a molecular devices plate reader (Spectramax M2; Molecular Devices, USA). One unit is defined as the amount of enzyme needed to exhibit 50% dismutation of the superoxide radical. Repeatability for 28 duplicate samples was 70.2% [58].

#### (vi) Glutathione

We assessed GSH level in RBC samples using the Cayman chemicals GSH assay kit (Cayman Chemical Company, USA). Similarly to the SOD assay, RBC samples were diluted in a 1 : 10 w/v solution using ice cold H_2_O MQ, then centrifuged at 10 000*g* for 15 min at 4°C. The supernatant was collected and deproteinated before the assay. The quantification is based on the detection of 5-thio-2-nitrobenzoic acid, produced by a reaction between the sulfhydryl group of GSH and 5,5′-dithio-bis-2-nitrobenzoic acid, using a molecular devices plate reader (Spectramax M2; Molecular Devices, USA). Repeatability computed for 37 duplicate samples was 87.9%.

#### (vii) Quantification of telomere length

Telomere length was determined using an established quantitative polymerase chain reaction (qPCR) protocol optimized for the banded mongoose (for details see [59]). In short, DNA was extracted from WBC using the DNeasy blood and tissue extraction kit (Qiagen) according to the manufacturer's instructions. Relative telomere length was determined as the ratio of telomere repeat copy number compared to the non-variable control gene (*M. mungo* inter-photoreceptor retinoid-binding protein gene, accession number AY170065) and standardized to a common (golden) sample run in parallel. PCR plate number was added as a random factor in analyses of telomere length to account for between-plate variation (estimated as 9% across plates).

#### (viii) Collection of faecal samples and quantification of faecal glucocorticoid metabolites

Faecal samples were collected during the morning latrine session soon after groups emerge from the burrow. Faecal samples were collected into small plastic bags, placed on ice and transferred to a −20°C freezer within 5 h of collection. To avoid interference with group scent marking behaviour, only half of each sample was collected. Hormone extraction, assay and validation was carried out at Chester Zoo Endocrinology Laboratory according to previously established and validated protocol [60,61].

### (d) Statistical analyses

We assessed the long-term consequences of maternal feeding during pregnancy with respect to physiological markers (oxidative stress, telomere length and faecal glucocorticoid metabolites) and fitness (start of reproduction, weight at maturity and LRS) of PRE and POST offspring. Linear mixed effects models (LMM) and generalized mixed effects models (GLMM) were used as detailed below. Additionally, for some analyses we compared offspring from experimental litters (PRE and POST offspring) with data on unmanipulated litters; all model results are included in full in the electronic supplementary material.

#### (i) Consequences of maternal supplementation on proxies of fitness

We measured *body mass at onset of adulthood, 12 months* of age (± 30 days). We computed a mixed effects LMM with body mass (in grams) as a response variable, and treatment group (PRE versus POST), sex and the interaction between treatment group and sex, as explanatory variables. Litter identity was included as a random effect. *Age at sexual maturity* (days) was assessed in two ways. First, the *age at first oestrus behaviour* (males: mate guarding females or ‘pestering’, i.e. approaching and trying to mate with females that are in oestrus; females: being mate guarded or pestered; e.g. [40]) was recorded from focal watch data, and used as an integer response variable in a Poisson mixed effects GLMM with social group, litter, and mother's identity as random factors. Individual identity was also fitted as an observation level random effect to account for overdispersion [62]. Second, *age at first successful reproduction* was deduced from parentage data of pups born after the onset of the study and used as the response variable in a Poisson model constructed the same way. *Survival* was monitored by visits to the group every 1–3 days. Banded mongooses disperse in groups, making it possible to unequivocally distinguish death from dispersal [63]. To study the effect of PRE and POST and sex on survival, we used a Cox mixed effects model as implemented in the package Coxme [64] with survival as the response variable, treatment group, sex and their interaction as explanatory variables, and maternal identity as a random factor.

Finally, we estimated LRS of individuals using the high quality population pedigree constructed earlier based on 43 polymorphic microsatellite markers [65,66]. Number of offspring assigned to the individual was the response variable in a Poisson GLMM, with treatment group, sex and their interaction as explanatory variables and social group, litter and maternal identity as random factors. Observation time, measured as the number of days the individual was alive between start of study and end of data collection for the pedigree analysis was included as an offset in the analysis, to account for potential differences in longevity, and for some of the individuals still being alive and potentially producing more offspring, after the last round of genotyping in 2019.

#### (ii) Consequences of maternal supplementation on offspring physiology

*Glucocorticoid metabolites*. Effects of treatment on offspring stress physiology during growth and across the lifespan was analysed using LMMs with faecal glucocorticoid content (ng g^−1^) as the response variable. The model looking at effects across the lifespan had treatment group, sex and their interaction, age at sampling, weight at emergence from the den (standardized, *z*-score), and social care during growth (escorting index, 0–1) as explanatory variables, and individual identity, litter, mother and social group as random factors. Model using samples during growth (age less than six months) had the same predictors but only litter as a random factor, to improve model convergence with lower sample size. Escorting index was calculated as the proportion of days that a pup was recorded with an escort, out of the total number of days where escorting was recorded by any group member (7–21 observation days per breeding attempt), and it was included in the analyses together with emergence weight to account for variation within treatment groups.

*Telomere length*. Potential effects of treatment on telomere length of offspring were assessed at first sampling point (median age at sampling = 38 days, range 1–9 months) with a LMM that included sex, treatment group (PRE versus POST) and their interaction, emergence weight and escorting index as above, and age at sampling as explanatory variables, and social group, litter, mother's identity and sample plate as random effects. Second, we analysed telomere length across the lifespan from repeated samples (average 2.4 samples, range 1–8 per individual, taken between the ages of one month to 4.7 years of age) with the same model, but also adding individual as a random term in the model.

*Oxidative stress markers*. We did not have oxidative stress measures for all markers for each individual, because of limited size of the blood samples, and thus we could not use data reduction methods such as principal components analysis to reduce the number of models. In any case, the markers of oxidative damage and antioxidant defences were not significantly correlated (all pairwise *r* < 0.2, *p* > 0.18). To examine whether the markers of oxidative damage and antioxidant defence were impacted by the provisioning experiment, we ran a set of LMMs, with oxidative stress markers as a response variable (either PC, MDA, GSH or SOD) and treatment group (PRE versus POST), age at sampling, emergence weight as above, sex and the interaction between treatment group and sex, as explanatory variables. Litter and pup identity were included as random effects.

Data were analysed using R, v. 3.6.1 (R Core Team 2017) using package lme4 to run linear and Poisson mixed-effects models [67] and package MuMIn [68] to calculate the marginal and conditional *R*^2^, i.e. variance explained by the models, with and without random factors. For all analyses, we checked model assumptions, i.e. normality, linearity and homoscedasticity. When required, the data were normalized by using a log-transformation (for GSH data) or square root (faecal glucocortiosteroids). We used likelihood ratio tests to assess the significance of variables. Non-significant interactions were dropped from final models to allow us to test the significance of the main effects included in these non-significant interactions [69]. Full details of the statistical models, test statistics for dropped interactions and random factors included in each are described in the electronic supplementary material.

*Power analyses*. As some analyses had limited sample size, we ran *post hoc* tests to investigate the statistical power of our analyses to detect a difference between treatment groups based on actual effect size. In addition, we calculated the power our analyses would have had, to detect a small (Cohen's *d* = 0.2) difference between the treatment groups. Calculations were done using G*Power [70] and actual analysis power is reported for all analyses comparing treatment groups.

## 3. Results

### (a) Life-history effects

#### (i) Weight at 1 year

At onset of adulthood, males were marginally heavier than females (weight at 1 year of age ± s.e.: 1293 ± 24.8 g, *n*= 35 versus 1226 ± 25.1 g, *n* = 15, *β* = 85.45 ± 44.12, $\chi_{1}^{2}=3.83,$ *p* = 0.050, but no differences in size remained between PRE and POST offspring (treatment group: $\chi_{1}^{2}=2.77,$ *p* = 0.096; marginal/conditional *R*^2^ = 0.056/0.967; statistical power = 0.92). There was no difference in weight between experimental and unmanipulated litters (litter type: $\chi_{1}^{2}=0.007,$ *p* = 0.934).

#### (ii) Age at first reproduction

Females showed behavioural signs of sexual maturity at a younger age than males (age at first observed oestrus behaviour ± s.e.: females: 320 ± 6.5 days, *n* = 17; males: 653 ± 60.06 days, *n* = 23; *β* = 0.613 ± 0.096, $\chi_{1}^{2}=44.95,$ *p* = 2.022 × 10^−11^) but there were no significant differences between PRE and POST offspring (treatment group: $\chi_{1}^{2}=0.143,$ *p* = 0.705; marginal/conditional *R*^2^ = 0.440/0.991, statistical power = 0.33), nor between experimental and unmanipulated litters (litter type: $\chi_{1}^{2}=0.01,$ *p* = 0.919). Similarly, females started breeding earlier (age at first successful reproduction: females 530 ± 29.4 days, *n* = 12, males 1011 ± 80.9 days, *n* = 13; *β* = 0.626 ± 0.094, $\chi_{1}^{2}=25.21,$ *p* < 0.00001) and there were no differences in the onset of reproduction between PRE and POST offspring (treatment group: $\chi_{1}^{2}=0.000,$ *p* = 0.997; marginal/conditional *R*^2^ = 0.653/0.991, statistical power = 0.05), nor between experimental and unmanipulated litters (litter type: $\chi_{1}^{2}=0.084,$ *p* = 0.772); but note that sample sizes are relatively low for these analyses, as is statistical power.

#### (iii) Survival

**Figure 2.**
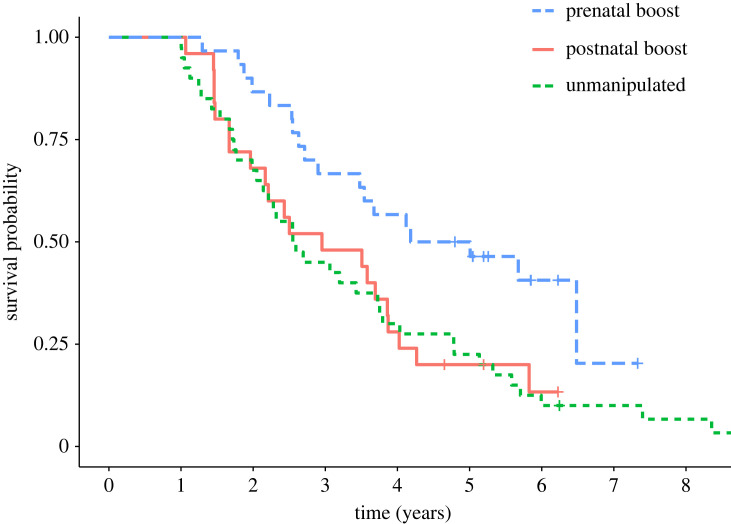
Effects of prenatal and postnatal investment on adult survival. Prenatal boost offspring had longer adult lifespan (survival past 1 years of age) than postnatal boost offspring, or those born in unmanipulated litters.

Prenatal boost offspring had higher adult survival than both POST offspring or those born in unmanipulated litters (treatment group: $\chi_{2}^{2}=8.16,$ *p* = 0.017, hazard ± s.e.: 0.43 ± 0.375, *z* = −2.25, *p* = 0.024, *n* = 95; figure 2). Specifically, from the age of 1 year (adulthood), PRE offspring survived at 2.3 times the rate of POST offspring. Median [maximum] lifespan of PRE versus POST offspring was 60 [88] and 44 [67] months, respectively. There was no difference in adult survival between males and females ($\chi_{1}^{2}=0.002,$ *p* = 0.96, hazard ± s.e.: 0.99 ± 0.258) nor sex-specific effects of treatment (sex:treatment $\chi_{2}^{2}=0.862,$ *p* = 0.65).

#### (iv) Lifetime reproductive success

**Figure 3.**
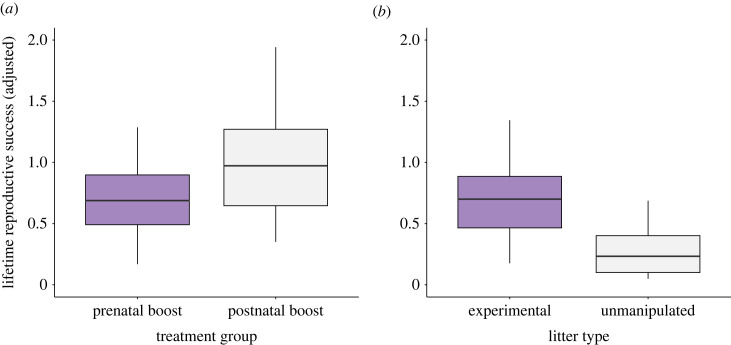
Effects of elevated prenatal and postnatal investment on lifetime reproductive success, adjusted for varying observation time (graph shows model predictions after controlling for differences in individual lifespan). Reproductive success was (*a*) higher for postnatal boost offspring than for prenatal boost offspring, and (*b*) higher in offspring born in experimental versus unmanipulated litters. Boxes show the interquartile range (IQR) and median (internal horizontal line). Whiskers (vertical lines) show the extent of observations outside the IQR to a maximum of 1.5× IQR.

Postnatal boost offspring had higher LRS than PRE offspring (*β* = −0.618 ± 0.246, $\chi_{1}^{2}=6.069\pm 0.014$; figure 3*a*). There was no sex-specific effect of treatment ($\chi_{1}^{2}=0.258,$ *p* = 0.611), but overall females had higher LRS than males (*β* = −1.397 ± 0.259, $\chi_{1}^{2}=29.38$, *p* < 0.00001, here calculated based on known offspring born in the monitored groups within the study area; marginal/conditional *R*^2^ = 0.300/0.357, analysis power 0.98), which probably reflects absence of information on male reproductive success gained outside the population (offspring were more likely to have an unknown father than a mother) [71]. Additionally, although the model did account for unequal observation time, males tend to reproduce later than females, and as some individuals were still alive and reproducing at the last round of parentage analysis done on the population, we probably have underestimated male reproductive success. Overall, pups born in experimental litters had higher LRS than pups born in unmanipulated litters (*β* = 1.55 ± 0.48, $\chi_{1}^{2}=10.69,$ *p* < 0.00001; figure 3*b*; marginal/conditional *R*^2^ = 0.309/0.397, analysis power=0.99).

### (b) Physiological effects

#### (i) Telomere length

Offspring that were heavier at emergence from the den had longer telomeres at first sampling point (*β* = 0.157 ± 0.065, $\chi_{1}^{2}=5.86,$ *p* = 0.019) but there were no effects of treatment group, sex nor age on telomere length (treatment group: $\chi_{1}^{2}=1.29,$ *p* = 0.263; sex: $\chi_{1}^{2}=0.885,$ *p* = 0.353, age at sampling: $\chi_{1}^{2}=1.45,$ *p* = 0.233; marginal/conditional *R*^2^ = 0.098 /0.643 and statistical power = 0.84). Across the lifespan, individuals that had been heavier as pups still had longer telomeres (*β* = 0.011 ± 0.048, $\chi_{1}^{2}=2.28,$ *p* = 0.028), but there were no effects of age, sex nor treatment group on telomere length (all *p* > 0.09, marginal/conditional *R*^2^ = 0.044/0.834, statistical power = 0.97).

#### (ii) Faecal glucocorticoid metabolites

**Figure 4.**
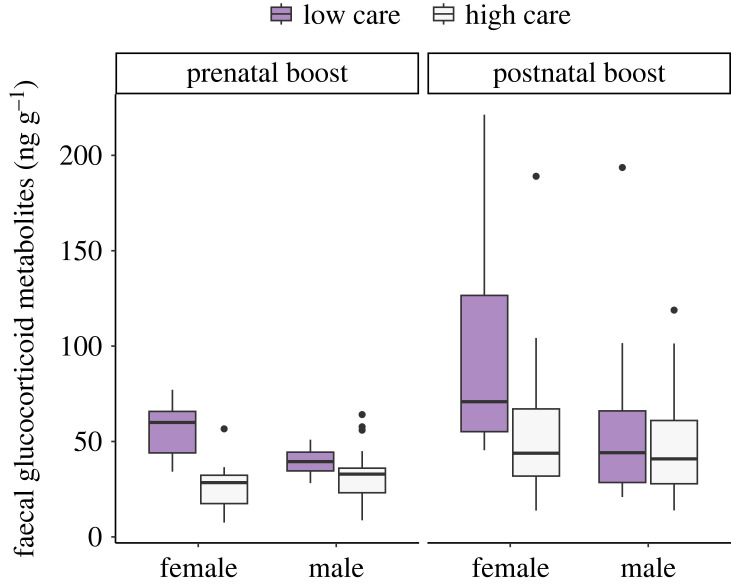
Effect of prenatal and postnatal investment on glucocorticoid levels across the lifespan. Faecal glucocorticoids across the lifespan (greater than six months) were lower in prenatal boost than in postnatal boost offspring, and lower in individuals that had received more social care as a pup. Escorting (proportion of focal sessions pup was escorted) was analysed as a continuous variable but here is split into high (>0.5) and low (<0.5) categories for illustration. Overall, female offspring had higher levels of faecal glucocorticoids than males. Boxes show the interquartile range (IQR) and median (internal horizontal line). Whiskers (vertical lines) show the extent of observations outside the IQR to a maximum of 1.5× IQR.

During growth (less than six months), PRE offspring had lower faecal glucocorticoid metabolite levels than POST offspring (*β* =−1.395 ± 0.431, $\chi_{1}^{2}=10.47,$ *p* = 0.0038, statistical power 0.88) and glucocorticoids decreased with increasing social care (*β* =−3.916 ± 0.783, $\chi_{1}^{2}=25.03,$ *p* = 0.00046) and increasing emergence weight (*β*= −1.222 ± 0.394, $\chi_{1}^{2}=9.596,$ *p* = 0.005) but there were no differences between males and females ($\chi_{1}^{2}=1.578,$ *p* = 0.224; marginal/conditional *R*^2^ = 0.549/0.833). Across the rest of the lifespan (greater than six months), PRE offspring still had lower levels of glucocorticoids as compared to POST offspring (*β* = −1.486 ± 0.346, $\chi_{1}^{2}=18.44,$ *p* = 0.00003), males had lower levels compared to females (*β* = −1.110 ± 0.358, $\chi_{1}^{2}=9.601,$ *p* = 0.0024), and the more escorting care individual had received as a pup, the lower its faecal glucocorticoid levels were across the lifespan (*β* = −2.723 ± 0.740, $\chi_{1}^{2}=13.57,$ *p* = 0.0004; figure 4). Glucocorticoid levels also increased with age (*β* = 0.724 ± 0.321, $\chi_{1}^{2}=5.36,$ *p* = 0.023; marginal/conditional *R*^2^ = 0.199/0.619; statistical power 0.99). When comparing individuals born in experimental litters to unmanipulated litters, there was a significant interaction between litter type and sex ($\chi_{1}^{2}=0.615,$ *p* = 0.0028): both males and females born in experimental litters had higher cortisol levels than those in unmanipulated litters (Tukey *post hoc* test, estimate ± s.e.: females 2.24 ± 0.4, *p* < 0.001, males 0.92 ± 0.36, *p* = 0.046), and whereas in experimental litters, males had lower glucocorticoids than females (estimate: −1.183 ± 0.390, *p* = 0.011), there was no overall sex difference in unmanipulated litters (estimate 0.350 ± 0.305, *p* = 0.632).

#### (iii) Oxidative stress markers

There were no significant differences between PRE and POST offspring in any of the oxidative stress markers (SOD: *β* = 54.699 ± 39.365, $\chi_{1}^{2}=1.930,$ *p* = 0.175, statistical power = 0.99; PC: *β* = 0.722 ± 4.98, $\chi_{1}^{2}=0.019,$ *p* = 0.890, power = 0.43; GSH: *β* = −0.077 ± 0.153, $\chi_{1}^{2}=0.257,$ *p* = 0.616, power = 0.39; MDA: *β* = 0.254 ± 0.175, $\chi_{1}^{2}=2.12,$ *p* = 0.154, power = 0.87). Males had lower levels of SOD (*β* = −130.96 ± 45.38, $\chi_{1}^{2}=8.32,$ *p* = 0.007) and borderline lower GSH (*β* = −0.344 ± 0.172, $\chi_{1}^{2}=3.97,$ *p* = 0.056) than females, but there were no sex differences in MDA ($\chi_{1}^{2}=0.899,$ *p* = 0.374) nor PC ($\chi_{1}^{2}=0.014,$ *p* = 0.908). Emergence weight was not associated with any of the markers (all *p* > 0.372; see data tables in the electronic supplementary material for detailed results). When comparing experimental to unmanipulated litters, there was an interaction between sex and litter type in SOD (*F* = 4.799, *p* = 0.334); the only significant pairwise difference was that experimental litter females had higher SOD than those in unmanipulated litters (Tukey *post hoc* test *z* = −2.716, *p* = 0.033). There were no differences between experimental and unmanipulated litters in any of the other markers (all *p* > 0.16).

### (c) Power analyses

According to *post hoc* power analysis, our probability to detect significant differences between treatment groups (i.e. actual statistical power to detect an effect, based on the size of potential treatment group effect and sample size) varied between 0.05 and 0.99. Average achieved power was higher in those analyses that yielded significant results (0.98 versus 0.62). However, the power to detect a moderate-sized effect (*d* = 0.2) was similar for significant and non-significant results (average power 0.822 for non-significant results, 0.854 for significant results), suggesting that non-significant results reported for the effect of treatment on certain life-history traits, telomere length and oxidative stress were not primarily a consequence of limited sample size. Nevertheless, the power analyses suggest that sample size may have been an issue in some analyses, for example, in our analysis of age at first oestrus behaviour; see the electronic supplementary material.

## 4. Discussion

When our study was conceived, its initial aim was to test the effect of manipulating maternal resources during pregnancy on future offspring development and life history. However, as a result of the remarkable ‘levelling’ behaviour of banded mongoose mothers—in which mothers that had been fed during pregnancy subsequently provided additional care and resources to the offspring of unfed mothers—our single experimental manipulation resulted in two treatment categories of offspring, both of which received greater levels of investment compared to offspring in ummanipulated litters, but at different phases of development. As a consequence our study provides rare longitudinal experimental evidence that prenatal and postnatal investment have distinct long-term effects on the survival, reproduction and physiology of offspring.

First, providing high quality food to pregnant mothers had large effects on adult survival of their offspring. Maternal provisioning increased the adult survival rate of offspring by a factor of 2.3 compared to the offspring of unfed mothers, and increased median adult lifespan by 36%. Second, offspring that received increased care and food during the postnatal period had higher LRS than the PRE offspring in the same litter, or offspring in ummanipulated litters. It is also notable that these POST offspring had higher levels of glucocorticoids across their lifetime than PRE offspring. Third, both categories of experimental offspring had higher LRS than offspring from unmanipulated breeding attempts. Thus the extra resources that we provided to half of the pregnant females in each banded mongoose group resulted in fitness benefits that were distributed across the whole communal litter, as a consequence of the cooperative care system of banded mongooses.

**Figure 5.**
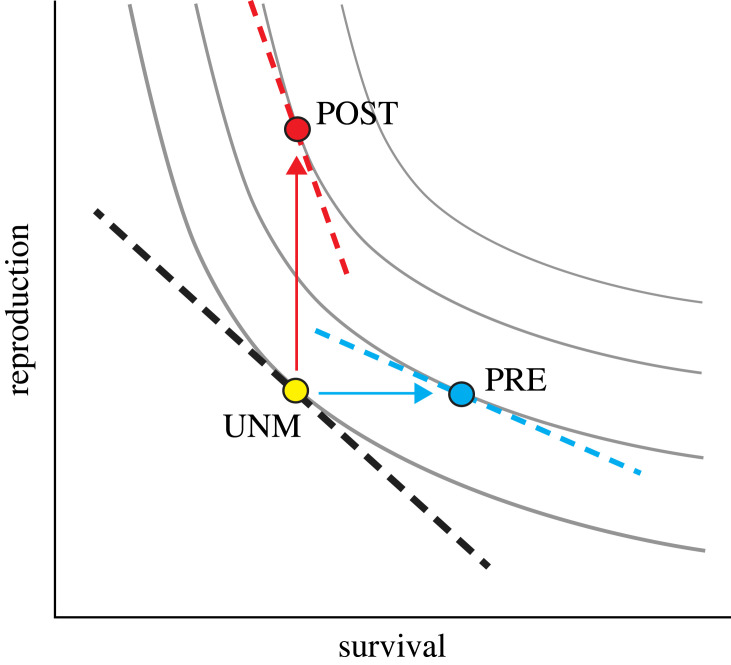
Graphical model of the effect of the experiment on optimal allocation of resources to reproduction (e.g. reproductive effort) and survival (adapted from [75] and [76]). Curved grey lines are fitness isoclines; points along each isocline represent allocations to reproduction and survival that yield equal fitness, with the fitness of isoclines increasing towards the top right corner. The black dashed line is the trade-off faced by unmanipulated offspring, assumed linear for simplicity. Given this trade-off, the optimal life-history allocation for unmanipulated offspring (UNM; yellow dot) is the point at which the trade-off is tangential to the fitness isocline. Offspring whose mothers were fed during pregnancy (PRE offspring; blue dot) exhibited higher survival than both UNM and POST offspring, higher fitness than UNM offspring, and lower fitness than POST offspring. This shift is consistent with prenatal provisioning attenuating the slope of the trade-off between survival and reproduction (blue dashed line). Offspring whose mothers were not fed, but who received additional care and resources during the postnatal period (POST), exhibited similar adult surviviorship to UNM offspring, but higher fitness than either UNM or PRE offspring (red dot). This shift is consistent with increased postnatal investment and faster postnatal growth steepening the slope of the trade-off between reproduction and survival. Similar changes in the slope of the trade-off between survival and reproduction are observed in response to fluctuations in postnatal ecological conditions in this species [76].

These results are consistent with the assumption of life-history theory that individual banded mongooses face a trade-off in the allocation of energy between survival and reproductive functions [72–74], and that extra investment in prenatal and postnatal periods can alter the shape and/or slope of this trade-off. A graphical model helps to visualize the nature of the shift in life-history strategy effected by the experiment (figure 5). Extra investment in prenatal and postnatal periods boosted the fitness of both types of offspring, consistent with the life-history assumption that resources limit the summed energy allocations between survival and reproductive functions. However, the extended lifespan of PRE offspring came with an associated reduction in reproductive output compared to POST offspring; the increased reproductive success of POST offspring came at the expense of lower survival in adulthood compared to PRE offspring. The graphical model suggests that these patterns can be explained if additional investment at prenatal and postnatal phases of development has contrasting impacts on the slope of the trade-off between survival and reproduction. Specifically, increased prenatal investment may lessen the slope of this trade-off, while increased postnatal investment may steepen it. This pattern is similar to the effect of natural variation in food availability during development on optimal scheduling of reproduction and survival across the lifetime in banded mongooses [76]. Males who experienced harsher (i.e. dryer) conditions during postnatal development lived longer lives but with reduced fertility; those who experienced more benign (wetter) conditions postnatally exhibited increased fertility but a shorter adult lifespan [76].

The finding that feeding mothers during pregnancy had dramatic impacts on the adult survival of her offspring represents rare experimental evidence for ‘foetal programming’ of adult health and fitness in a wild mammal. Fed mothers gave birth to heavier offspring [45], a metric that is associated with increased lifespan in humans [77]. Maternal under-nutrition and low birth weight in both humans and animal models is associated with negative health outcomes including increased risk of cardiovascular and metaboloic diseases [78,79]. On the other hand, maternal over-nutrition in the form of maternal obesity and high fat/high sugar diets also has deleterious effects on offspring health and lifelong susceptibility to obesity and cardiac disease [80,81]. It is usually difficult to assess, however, how health outcomes in humans and model laboratory organisms relate to impacts of early life conditions on individual fitness. Assessing the fitness consequences of early life effects is vital to understand how natural selection can be expected to shape the genetic and epigenetic developmental mechanisms underlying these patterns of fetal programming and plasticity. Our study shows that a relatively short perturbation in maternal nutrition in pregnancy (supplemental provisioning with a high value natural component of the diet, leading to a short-lived increase in maternal body mass [45]) altered the pattern of fetal growth in a manner that yielded very large survival benefits in adulthood and increased LRS compared to unmanipulated offspring. These results are strong evidence that the observed pattern of fetal programming in banded mongooses is adaptive, in the sense that offspring were able to use the availability of extra maternal resources to increase their lifetime fitness [30]. Moreover, the effect of prenatal nutrition on adult survival emerged despite there being no difference between treatment groups in body mass at adulthood. Maternal feeding in the latter half of pregnancy could potentially induce prenatal changes in organ growth, physiology or the capacity for learning in a way that has major survival impacts in adulthood without any detectable impact on overall size or body mass at adulthood.

The second finding that POST offspring exhibited increased LRS suggests that adult fitness is particularly sensitive to the amount and quality of food and care received in the postnatal period after emergence and up to nutritional independence. Escorts carry, groom, and protect offspring, help them to find food, teach them specialized foraging techniques [82], and pass on food niche preferences [42]. The increased postnatal investment received by the offspring of unfed mothers was more than enough to overcome their initial disadvantage in birth weight and translated into higher LRS despite these individuals living shorter lives in comparison to PRE offspring (but not unmanipulated offspring). These experimental findings add to previous evidence from long-term data showing that offspring who receive more escorting have higher LRS, over and above any effect on body size [5]. Again we can speculate that postnatal investment by escorts may change organ growth and physiology of offspring, or provide cultural training [42] in a way that increases the efficiency with which they are later able to convert resources into surviving offspring.

The sensitivity of fitness to postnatal investment in mammal societies highlights the inclusive fitness benefits that escorts stand to gain from guarding and provisioning offspring to independence. In banded mongooses synchronous birth appears to remove the ability of parents or helpers to discriminate relatedness of pups in the communal litter, such that in natural litters escort-pup pairs are no more closely related than expected by chance [45]. Nevertheless, average relatedness among group members is around 0.2, with almost no migration between extant groups and strong genetic population structuring (*F*_ST_ among groups = 0.13; [83]), meaning that indiscriminate helping can in theory bring substantial kin selection (and/or group selection; [84,85]) benefits. In our experiment, helping was not indiscriminate, it was *anti-nepotistic,* since fed mothers targeted their helping effort to offspring to whom they were related by median relatedness 0.09, whereas mean relatedness of fed mothers to all offspring in experimental litters was 0.18 [45]. Evolutionary game theory models show that distributing care to those in most need can be an inclusive fitness maximizing strategy from behind a veil of ignorance about the genetic returns on investment [45]. Targeting of investment according to need is further strengthened if the developmental linkage between prenatal and postnatal stages is such that postnatal investment can compensate or overcompensate for early life disadvantage. Our experiment shows that escorting behaviour can compensate fully in terms of adult weight for early life disadvantage in offspring birth weight, and indeed can result in elevated LRS compared to offspring that receive less escorting.

Our study failed to detect significant differences between treatment groups in our measures of oxidative stress and telomere length. Contrary to our expectation, mongoose pups that faced early life disadvantage did not show increased oxidative stress nor shorter telomere length, as would be expected if the catch-up growth that they experienced came at a cost to cellular maintenance (e.g. [46]). Other studies in wild mammals have found similar results (e.g. [86]), namely, that accelerated growth was not associated with oxidative cost or telomere loss, potentially owing to individual plasticity. The lack of significant links between life-history patterns and oxidative stress in our analyses may be owing to variety of factors, including unaccounted for covariates, limited sample size, and selective disappearance. Indeed, a previous study on our system suggested that females selectively abort pups when faced with a potentially harsh post-birth environment [87]. Such patterns of early life mortality, long before we are able to physiologically sample pups that emerge from the den, are likely to further obscure any links between physiological measures and life-history traits. Nevertheless a key finding of our physiological investigation was that the elevated catch up growth of POST offspring was associated with elevated glucocorticoids across the lifespan. This finding adds to evidence in wild vertebrates that glucocorticoids (and, potentially, telomere lengths) play an important role in mediating changes in the trade-off between survival and reproduction [49], such as the trade-off shifts illustrated in figure 5. If replicated in a population health or biomedical context, this effect could have health and disease implications.

Our experiment did not provide evidence for the developmental mismatch hypothesis [28,29,88,89], since both PRE and POST offspring experienced a mismatch between the quality of pre- and post-natal rearing environments, yet both exhibited higher LRS than unmanipulated offspring. However, an important caveat is that we were limited to investigating only a ‘positive’ type of mismatch, involving a boost to one stage of development, rather than a ‘negative’ mismatch involving particularly harsh conditions. Rather, our prenatal and postnatal investments combined to shape future fitness and life history in the manner modelled using Heckman functions in skill formation theory [18,19,21]. Heckman functions are mathematical expressions in which investment at one stage of development can influence offspring ‘capital’ (or fitness) in two ways, termed ‘self-productivity’ and ‘complementarity’ [21]. Self-productivity captures the idea that the effect of investments in offspring persist over time: offspring who received more investment at an early stage have a platform on which future investments can build. Complementarity captures the idea that investment at an early stage increases the effectiveness of investment at later stages in raising fitness: offspring that have received more investment can make better or more efficient use of investment at a later stage. Both types of influence are closely related to what in the biological literature are called ‘silver spoon effects’ [90]. In humans, patterns of self-productivity and complementarity are such that attempts remediate in adolescence for early life disadvantage are very costly, whereas interventions targeted at early childhood are much more effective [21]. Skill formation studies [18,21] have focused on the nature of complementarity between postnatal investments, whereas less is known about the interacting effects of investments in prenatal and postnatal stages (but see [25,91]). In an evolutionary context, we might expect self-productivity and dynamic complementarity to coevolve with the amount of investment parents (and alloparents) are able to provide at each stage of development, and the degree of competition among offspring for those investments, two aspects of the rearing environment that may be very different in prenatal and postnatal periods.

The observed complementarity between prenatal and postnatal stages of investment in banded mongooses is similar to that found in a cross-fostering experiment on wild great tits (*Parus major*) in Sweden ([35]; see also [92] for a second factorial experiment in burying beetles). In the great tit experiment, some mothers received a food supplement in the prenatal period, equivalent to a PRE treatment. After hatching, offspring were partially cross fostered with some offspring receiving supplemental food as nestlings, equivalent to a POST treatment. Prenatal boost offspring were heavier at hatching than control offspring, but postnatal feeding of control offspring compensated for this initial size disadvantage, so that there was no mass difference at fledgling between those who had received either a PRE or a POST. In this experiment there was no evidence that a mismatch between prenatal and postnatal rearing conditions had a deleterious impact on offspring development. Unlike our experiment, however, this study could not follow long-term or lifetime effects of manipulated prenatal and postnatal investment, owing to low recruitment of offspring into the adult population. Our findings suggest that provision of supplementary resources during prenatal and postnatal periods can have dramatic effects on life history and fitness that are not detectable in measures of body mass or size at independence, and which may remain hidden in shorter term studies.

## 5. Conclusion

Our experimental study on a wild social mammal shows that the amount of resources and care received during prenatal and postnatal periods combine to mould the lifetime trajectories of survival, reproduction, and physiology of adults. As in humans [23,26], the level of investment at an early stage of development may influence the responsiveness of offspring to investment at later stages, leading to variation in adult life history. Remediation in the postnatal period can more than compensate, in fitness terms, for prenatal inequality, most likely by altering the shape of the trade-off between survival and reproduction in adulthood, albeit with potentially adverse consequences for stress physiology. Our results suggest that additional prenatal investment flattens this trade-off, whereas additional postnatal investment steepens it. To understand why prenatal and postnatal investments have distinctive impacts on development and life history may require improved evolutionary models of social development, perhaps adapted from models of skill formation [18,19], together with fully factorial experimental designs (e.g. similar to [35] and [92]) to reveal the lifetime effects of prenatal and postnatal investment and inequality.

## Data Availability

Supporting data for all analyses are available from the Dryad Digital Repository: https://doi.org/10.5061/dryad.d51c5b07m [93]. The data are provided in the electronic supplementary material [94].
